# A virtual clinic for the management of diabetes-type 1: study protocol for a randomised wait-list controlled clinical trial

**DOI:** 10.1186/s12902-020-00615-3

**Published:** 2020-09-05

**Authors:** Elisabet Nerpin, Eva Toft, Johan Fischier, Anna Lindholm-Olinder, Janeth Leksell

**Affiliations:** 1 Department of Medical Sciences, Respiratory Medicine, Allergy and Sleep, Uppsala University, Uppsala, Sweden; 2 Department of Medical Sciences: Clinical Physiology, Uppsala University, Uppsala, Sweden; 3 School of Education, Health and Social Studies, Dalarna University, SE-791 88 Falun, Sweden; 4 Department of Medicine, Huddinge, Karolinska Institutet, Stockholm, Sweden; 5 Diabetes Unit, Ersta Hospital, Stockholm, Sweden; 6 Department of Medical Sciences, Clinical Diabetology and Metabolism, Uppsala University, Uppsala, Sweden; 7 Department of Clinical Science and Education, Karolinska Institute, Södersjukhuset, Stockholm, Sweden

**Keywords:** Type 1 diabetes, Young adult, Virtual clinic, E-health, Randomised controlled trial

## Abstract

**Background:**

Diabetes is a serious chronic disease. Medical treatment and good psychosocial support are needed to cope with acute and long-term effects of diabetes. Self-management is a large part of diabetes management, with healthcare providers playing a supportive role. Young adults with type 1 diabetes are of special interest as they tend to have higher mean glycosylated haemoglobin values than other patients with type 1 diabetes, and they often miss visits in traditional diabetes care. A well-designed virtual solution may improve a range of measures (e.g. glycaemic control and perceived health) and reduce hospitalisations.

**Method:**

This randomised controlled trial with a control group using a wait list design will recruit 100 young adults from a hospital in Sweden. All participants will receive usual diabetes care besides the virtual clinic. The primary objective is to evaluate the effect of a virtual diabetes clinic on glycaemic control, treatment satisfaction and quality of life in young adults (aged 18–25 years) with type-1 diabetes. The secondary objective is to determine the effects of virtual care on the patient experience.

**Discussion:**

Virtual tools are becoming increasingly common in healthcare; however, it remains unclear if these tools improve diabetes self-management. The results of this study will build understanding of how healthcare providers can use a virtual clinic to improve diabetes self-management.

**Trial registration:**

Current controlled trials: ISRCTN, number: 73435627, registered 23 October 2019. 10.1186/ISRCTN73435627

## Background

Diabetes is a common and serious chronic disease that may have severe complications and is a cause of premature death [1, 2]. Diabetes is an ever-present condition characterised by a constant struggle and by feelings of constraint, of fear, shame and of being alone, but also by courage, by a sense of trusting one’s own abilities and by closeness with family, friends and others with diabetes [3, 4].

Self-management could be challenge and difficult to maintain for many diabetes patients. Patients describe their everyday life as a balancing act between high and low blood sugar concentrations, between diet and exercise and desire and demands [3, 4]. It is possible to live a good life with diabetes, but this is demanding. Self-management is a crucial part of diabetes management, especially for those with insulin treatment. Data obtained from the National Diabetes Registry in Sweden showed that many people with diabetes have difficulty achieving the recommended glycaemic control; in particular, young people find it difficult to manage their daily treatment requirements to achieve good blood glucose values [5, 6]. The reason for this is partly unknown; factors such as genetic variations, fear of hypoglycemia, stress, depression and lack of knowledge may be contributing factors [3, 7–9]. In addition, studies have shown that depression is two to four times more common in people with diabetes compared with the general population [10]. The self-management is often unsatisfied with many missed doses to meals and snacks [11].

Professional diabetes care is crucial for the patient’s ability to manage their diabetes. This is facilitated through medical and technical guidance and support according to the individual’s needs [12]. Through the rapid development of advanced treatment technology, higher demands are placed on diabetes caregiver in order to provide care, education, and support for patients with diabetes and their relatives [13]. Today, the number of personal diabetes visits is increasing and in combination with a lack of glycemic control and advanced diabetes technology, it often creates an overwhelming burden for traditional diabetes care [5, 6, 14]. The roles and responsibilities of healthcare professionals have also changed from one who ‘tells’ to one who ‘listens’, which highlights person-centred care compared with a conventional hierarchical approach [12]. This raises the question as to whether the diabetes care currently offered to patients living with diabetes is optimal.

Previous studies in this area have evaluated components of virtual clinics (e.g. mobile applications, SMS and computer-based diabetes care) as part of telemedicine. However, these studies did not focus on patient-centred virtual diabetes care. A recent literature review focused on telemedicine for diabetes that included 55 randomised controlled trials found that compared with conventional diabetes care, telemedicine improved treatment outcomes for patients with type-1 and type-2 diabetes [15]. That review found slightly better results for patients with type-2 diabetes; however, glycosylated haemoglobin (HbA1c) was reduced in patients with type-1 and type-2 diabetes. The discussion of the limitations of the review indicated that as the measure used was HbA1c, potential for improvements in other areas of diabetes management were not assessed (e.g. fear of hypoglycaemia or satisfaction with treatment). In another review focusing on type 1 diabetes and distal technologies (various electronic systems providing remote services) *telehealth*, such as telephone calls and video appointments, was the one technology that hitherto could prove similar or better glycaemic control with potential additional benefits, regarding costs, efficiency geographic barriers and convenience [16]. The need to assess other health parameters in addition to HbA1c, as well as conducting studies with higher methodological quality has been confirmed by other research [17, 18]. Moreover, previous research has not focused on the difficult group of young adults with type 1 diabetes. This group of patients is of special interest to try to reach with virtual diabetes care, as they have higher mean HbA_1_c values than other patients with type 1 diabetes. In addition, they often miss visits in traditional diabetes care. At the same time, they frequently have advanced technologies (e.g. insulin pumps and continuous or flash glucose monitoring systems), which provide in-depth data, able to share on-line with health care professionals, allowing detailed analysis and advice for diabetes care at a distance.

A potential low-cost intervention to support diabetes self-management could be through virtual diabetes clinics via a smartphone app. Virtual clinics increase the opportunities for people with diabetes to retain contact with” their” diabetes nurse or doctor, allowing for continuity of care and providing a sense of security for many patients. Principles specific to virtual diabetes care are that patients can communicate from their home environment and if needed, have opportunity for more frequent contact with healthcare providers using virtual clinics.

### Study aim

The primary objective of this study is to evaluate the effect of a virtual diabetes clinic on glycaemic control, treatment satisfaction and quality of life in young adults (aged 18–25 years) with type-1 diabetes. The secondary objective is to determine the effects of virtual care on the patient experience.

## Method/design

### Study design

This study will be a randomised controlled trial with a control group according to a wait list design.

### Participants and recruitment

The inclusion criteria for the study are people: with type 1 diabetes; aged 18–25 years; and registered at a single hospital in Stockholm, Sweden. Participants will be identified from the diabetes clinic patient register by the hospital staff. Exclusion criteria are duration of diabetes shorter than 1 year, a diagnosis of severe depression; eating disorders or other serious mental illness; alcohol/drug abuse; or severe diabetes complications. The diabetes nurse and/or physician will be the person who makes the decision whether the diabetic patient has compliance to be able to participate in the study. The study hospital’s outpatient diabetic reception is currently responsible for about 410 patients in the target age group. Written and verbal information about the study will be provided to patients who meet the above criteria. Written informed consent will be obtained from all participants.

Participants who meet the inclusion criteria, are willing to take part in the study and provide informed consent will be randomly allocated to either the intervention group or a wait-list control group. The primary outcome/end point will be glycaemic control; HbA1c, Time In Range and Time Below Range, Severe Hypoglycemia and Diabetic Ketoacidosis, respectively. Secondary outcomes will be treatment satisfaction, quality of life and patients’ experience of virtual care. All participants will receive usual diabetes care in addition to the virtual clinic.

### Randomisation

After participants are identified, they will be randomised to an intervention group or a wait-list control group using closed randomisation envelopes containing randomisation cards. The closed envelopes have been developed by a person who is not involved in the inclusion or care of the patients.

All material is coded with consecutive digit code. The study manager will complete sealed envelopes containing randomisation cards. The nurses in the clinic will be responsible for the inclusion, randomisation, and the interviews. All personnel in the clinic will be informed about the study and trained to include subjects, collect data and the procedures to be conducted at each visit. The envelopes will be numbered from 1 to 100. The hospital staff will then take the top envelope when including a patient. All data will then be collected by existing staff at the relevant hospital. All material is coded with consecutive digit code. The code list is stored in a locked fireproof cabinet. A flow chart of the study is shown in Suppl. 1.

### Intervention

Baseline data will be collected for both groups before randomisation. The intervention group will be offered access to the virtual clinic instantly, and the wait-list control group will be offered the intervention after 6 months. At the 6-month follow-up assessment, a second data collection will be carried out for both groups (intervention and the wait-list groups). The intervention group will then continue with the intervention while the wait-list control group starts the intervention. The third data collection point for both groups will be after 12 months. Finally, data at 18 months will be collected for the wait-list group (after having access to the virtual clinic for 12 months).

Participants who meet the inclusion criteria will be randomly offered the chance to take part in the virtual clinic either immediately or after 6 months. Vista Dialog offers a digital service specially designed to facilitate continuity of care between diabetes patients and their nurses or doctors.

Vista Dialog supports a way of working that allows more frequent communication when needed, whilst allowing continuity of care and minimal disturbance to daily life. It provides additional access to healthcare without over- burdening health care professionals. For users, the virtual care increases the feeling of being in control and is easy to use and understand. The ability to provide instantaneous feedback means a more effective evaluation of health care interventions is possible. The simple method of facilitating further discussion contributes to an increased participation in the care which means that any potential problems or obstacles are discovered more quickly. Vista Dialog complements the usual care at the outpatient clinic and other eHealth services.

The virtual package/care consists of a platform partly delivered via a mobile application for patients and partly through a web interface/portal for staff. There is a secure log-in via a bank id system for patients and a secure login card for staff. Vista Dialog was developed in close collaboration with young people with diabetes and the platform allows both parties to easily communicate in real time via text messages. In addition, patients can book an online appointment with the Diabetes Specialist Nurse in times made available by the nurse via the application. It is also possible to start a spontaneous video meeting if a need arises in connection to a text message chat between patient and nurse. Documents or photos can easily be shared by patient or caregiver in the platform. Data from proximal technology (insulin pumps and CGM) is uploaded by the patient and can be reviewed and discussed in collaboration. Patients and caregiver have simultaneous access to data in platforms such as Diasend® or CareLink®. All together this invites and enables the patient to put forward their needs in the moment they arise. That contrasts to the conventional clinic where the patient needs to wait for a telephone consultation or the booked consultation in the future. Participants are well informed that the virtual clinic is not intended for very acute cases, such as ketoacidosis, and that the caregiver is only available during daytime weekdays.

### Measures and data collection

All data will be collected in-person at the diabetes clinic (clinical variables and psychometric measures). Outcomes measurement will be completed at baseline, 6 months, 12 months, and after 18 months in the wait-list group (control group).

#### Clinical variables

- Glycosylated haemoglobin (HbA1c) as an indication of glucose control. Measure time in range (3.9–10 mmol/L) and time below range through Continuous Glucos Monitoring (CGM) /Flash Glucose Monitoring (isCGM) [19].
- Insulin requirements/dosage to explore changes as a further indicator of glycaemic control, as missed doses are common [11]. At the standard clinic appointments, insulin dosage will be downloaded using diabetes management software (e.g. Diasend®).
- The total daily insulin dose will be recorded where possible.
- Type of treatment to describe if the patient uses multiple daily injections (MDI) or continuous subcutaneous insulin infusion (CSII).
- Diabetes duration and age at onset of diabetes (data collected at baseline).
- Sociodemographic data to describe gender, age, living at home/independent living and level of education (data collected at baseline).

#### Psychometric measures

- Diabetes Treatment Satisfaction Questionnaire: status version (DTSQs) and Diabetes Treatment Satisfaction: change version (DTSQc) (data collected after 12-months in the intervention group and after 18-months in the wait-list group (control group) (Information about DTSQs and DTSQc available at www.healthpsychologyresearch.com)
- Check Your Health
- Qualitative interview

##### Psychometric measures

In this study, we plan to use both DTSQs and DTSQc. It is common that researcher who wants to evaluate patient satisfaction in different diabetes treatment interventions use DTSQs in its original form (e.g. ‘How satisfied are you with your current treatment?’ Response options: very satisfied to very dissatisfied) [20]. However, surveys who evaluate patient satisfaction tend to produce small variations and most response express positive satisfaction. This leaves small or no rooms to show improved satisfaction later in the trial in respondents who previously scored at or near ceiling. A change version of the DTSQ (DTSQc) was therefore designed to overcome the ceiling effects found with the status version (DTSQs) [21].

The DTSQ encompasses three areas: general treatment satisfaction, hyperglycaemia, and hypoglycaemia. The questionnaire includes eight questions: six about different aspects of treatment satisfaction and two about hyper/hypoglycaemia that has occurred during the last few weeks. Responses are on a 7-point scale. DTSQs scores range from 0 = *very dissatisfied* to 6 = *very satisfied*, and DTSQc scores from ‘+ 3 = *much more satisfied now* to − 3 = *much less satisfied now*, with 0 (midpoint) representing no change’ [22]. The total score is the sum of all points from the questions about different aspects of treatment satisfaction. Therefore, increases in treatment satisfaction produce positive values and decreases in treatment satisfaction negative values, with 0 representing no change. For perceived hyperglycaemia and hypoglycaemia, positive scores indicate an increase in perceived hyperglycaemia or hypoglycaemia, and therefore deterioration in these outcomes [22].

### Check your health

‘Check your Health’ screens perceptions and experiences of physical and emotional health, social relationships, and general quality of life via a vertical thermometer scale. Responses range from 0 to 100, where 0 indicates a perceived very low level of health and quality of life and 100 a very high perceived level of health and quality of life. Using the same scale, a person then reports what they think their physical and emotional health, social relationships and quality of life would be if they did not have diabetes. When the difference is a positive value (e.g. that physical health without diabetes is reported as lower than with diabetes) the burden is interpreted as zero. Check your Health has been tested with both adults and young people with diabetes, and has been shown to have good reliability and validity [23, 24].

### Qualitative interviews

Qualitative interviews will be conducted with approximately 16 individuals. We will perform purposeful sampling with a maximum variation. The interviews will be conducted before and at the end of the intervention and follow a semi-structured interview guide. The interview guide will focus on the following three major topics, Suppl. 2.

- Perceptions about virtual care; for example, ‘Do you have any experiences of virtual care’ (before intervention), ‘Which changes do you see the virtual care has brought?’ (after intervention).
- Hindering and facilitating factors in the context of virtual care; for example, ‘Is it possible to describe some factors or events that may hamper virtual care’ (before intervention) and ‘Which conditions in your daily life have made it difficult or easy to achieve positive outcomes from the virtual care?’ ´Can you describe how you used the application (app) in your daily life and specific aspects of the app that were helpful/not helpful for you´ (after intervention).
- Problems with virtual care technology; for example, ‘Do you have experience in digital or virtual technology’ (before intervention) and ‘Did you have any problems with the internet or in reaching the diabetes team’ (after intervention).

### Statistical analysis and power calculation

To detect a mean difference of 6 mmol/mol (standard deviation [SD] = 9) in HbA1c, it will be necessary to include at least 37 participants in each group (alpha 0.05, power 80%, two-sided test). Taking dropout rates into consideration, a total of 100 patients will be included in the study. Participants will be randomly assigned to either experimental or control group with a 1:1 distribution as per a computer-generated randomisation.

All analyses will be conducted on an intention-to-treat basis. The difference at the 6-month follow-up between the intervention and wait-list control groups (adjusted for values at baseline) will be analysed with a two-sided independent samples Student’s *t*-test with a 95% confidence interval. A *p*-value < 0.05 will be considered statistically significant. Mean (SD) values and 95% confidence intervals will be used to describe the sample. A secondary analysis of the primary endpoint will adjust for baseline characteristics. For independent samples, a Student’s *t*-test will be used when comparing two categories for interval or ratio data, and a Mann-Whitney U test will be used for ordinal data. When three or more categories are compared, analysis of variance will be used for interval or ratio data and the Kruskal-Wallis test will be used for ordinal data. Generalised estimating equation regression models will be used for comparisons within groups over the three time points. All statistical analyses will be performed with IBM SPSS Statistics.

### Qualitative analysis

The text from the interviews will be analysed using qualitative content analysis [25, 26], a method that is suitable for obtaining inferences from verbal and communication data.

An experienced transcriber transcribes the recorded interviews. The entire research group then reads the transcribed text, to get an overall sense of the texts from the interviews. The analysis consists of a manifest and a latent phase.

- The manifest phase begins with a discussion regarding the transcribed text with all authors and divide into units of meaning, (corresponding to the aim). Three of the authors (EN, JF and JL) perform the operational work of selecting units of meaning. The entire research group then meets to crisscross that the condensed units of meaning are consistent with the aim.
- Once consensus on the units of meaning has reached, they are provided with a code. Two in the research group (EN and JL) conveys the different codes together, regarding similarities and differences. After discussion, all authors will agree that saturation has been reached (according to the codes).
- The codes will then be sorted into subcategories. The formation of these subcategories is done throughout the research group.
- The subcategories then form a basis for the main categories. These main categories are discussed throughout the research group. The formation of main categories is part of the manifest phase. The latent phase is an overall heading of the main categories.

## Discussion

The aim of this study is to evaluate the effect of a virtual diabetes clinic on treatment satisfaction, quality of life and glycaemic control in young adults (aged 18–25 years) with type-1 diabetes. We chose to include young adults because we want to see if participants using virtual diabetes care show improved: HbA1c levels; time in ranges for continuous subcutaneous glucose monitoring; quality of life; physical, mental and social health; and general treatment diabetes satisfaction. Time in range is the percentage of *time* that glucose levels are in low, target and high *ranges*. We will use Check Your Health Scale and treatment satisfaction assessed with the DTSQ, to detect changes in perceived health and general quality of life. The evaluation points for the intervention will be at baseline, and 6 and 12 months (virtual care) to allow comparisons and determine any changes.

In Sweden, all Patients with type 1 diabetes are treated at specialist centres, which means that all patients at the clinic who fulfil the inclusion criteria can be enrolled, regardless of race and ethnicity.

Data from national register in Sweden, include almost 90% of all diabetes patients. Data indicate that more than 70% of young patients with type 1 diabetes have a HbA_1_c level above the target level of 52 mmol/mol [27]. For this reason, it is important to take actions to support patients to attain optimal glycaemic control and at the same time maintain a good quality of life and to postpones diabetes-related complications.

The potential reduction of unsatisfactory glycaemic control and burden of diabetes using virtual diabetes care would be of benefit, and potentially even more clinically significant, in comparison with usual care (i.e. face-to face care meeting).

### Study limitations

We can see some limitations for our study. First, we only include patients from one clinic, which may limit the generalizability. The admission area for the study hospital comprises an entire county and thus includes patients with a mixed socio-economic background, which increases the external validity. Secondary, young adults can be a difficult age group to include. However, the clinic is responsible for approximately 400 young adults with type 1 diabetes, so we do not think that this will affect our inclusion to the study.

## Supplementary information

**Additional file 1:** **Suppl. 1.** Flow chart virtual diabetes clinic with waiting list design. Flow chart virtual diabetes clinic. Patients of the wait list control group will be offered virtual diabetes clinic after 6 months.

**Additional file 2:** **Suppl. 2.** Interview guide.

## Data Availability

Data sharing is not applicable to this article, as no datasets were generated or analysed for this study protocol.

## Abbreviations

CGM: Continuos Glucose Monitoring
isCGM: Continuous Glucose Monitoring with intermittent scanning / Flash glucose monitoring
CSII: Continuous Subcutaneous Insulin Infusion
DTSQ: Diabetes Treatment Satisfaction Questionnaire
DTSQc: Diabetes Treatment Satisfaction:change version: change version
DTSQs: Diabetes Treatment Satisfaction Questionnaire: status version
FGM: Flash Glucose Monitoring
HbA_1_c: Glycosylated haemoglobin
MDI: Multiple Daily Injections
SD: Standard Deviation
