# Supplementary figures and images for: A virtual clinic for the management of diabetes-type 1: study protocol for a randomised wait-list controlled clinical trial
Source: BMC Endocr Disord. 2020 Sep 5;20:137. doi: 10.1186/s12902-020-00615-3 (PMC7487575; doi:10.1186/s12902-020-00615-3)

Supplementary file 1. Flow chart virtual diabetes clinic with waiting list design

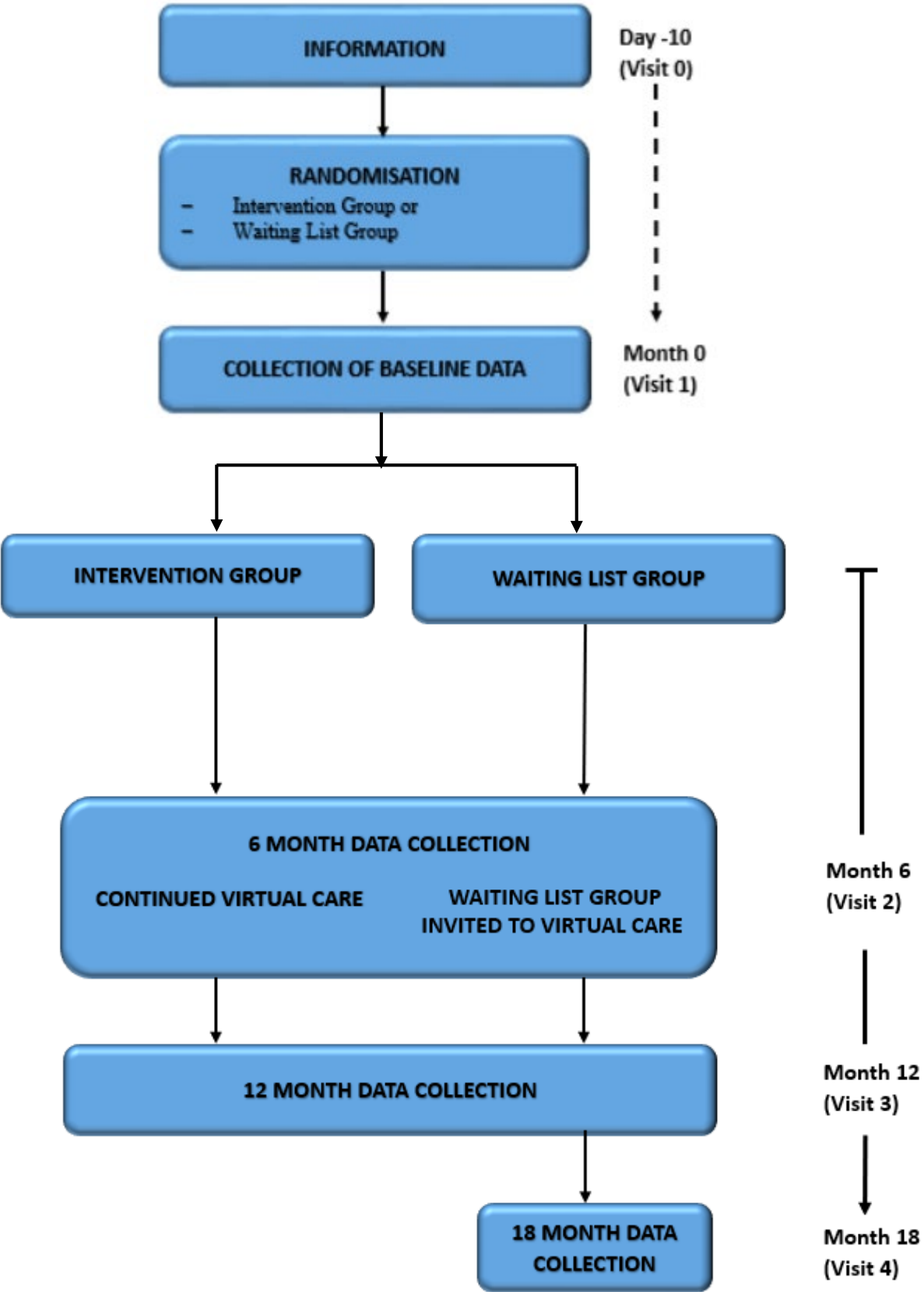

Supplement: Supplementary file 1 — Additional file 1: Suppl. 1. Flow chart virtual diabetes clinic with waiting list design. Flow chart virtual diabetes clinic. Patients of the wait list control group will be offered virtual diabetes clinic after 6 months. [file 12902_2020_615_MOESM1_ESM.pdf]
