# Supplementary material for: A virtual clinic for the management of diabetes-type 1: study protocol for a randomised wait-list controlled clinical trial
Source: BMC Endocr Disord. 2020 Sep 5;20:137. doi: 10.1186/s12902-020-00615-3 (PMC7487575; doi:10.1186/s12902-020-00615-3)
Supplement: Supplementary file 2 — Additional file 2: Suppl. 2. Interview guide. [file 12902_2020_615_MOESM2_ESM.pdf]

Interview guide

- Perceptions about the virtual care (e.g." Do you have any experiences of virtual care" (before intervention) "Which changes do you see the virtual care has brought?"(after intervention>).
- Hindering and facilitating factors in the context of virtual care (e.g. "it is possible to describe some factors or events that may hamper virtual care" (before intervention) "Which conditions in your daily life have made it difficult or easy to achieve positive outcomes from the virtual care?"(after intervention).
- Problem with virtual care technology (e.g. "Do you have experience in digital or virtual technology" (before intervention) "Did you have any problems with the internet or in reaching the diabetes team" (after intervention)

People with experience of individual interviews and diabetes care will perform the interviews. Interviews about 1-1.5 hours. All interviews are recorded on tape recorder and transcribed. An interview guide is prepared, in addition, follow-up questions will be asked of the type:

- can you give a concrete example?
- can you develop that reasoning?
- what was it that made you feel or think that way?

Before the Intervention

1. *How do you feel and think when introducing Virtual Meeting in Diabetes Care?*
2. *What experience do you have from your meetings at the diabetes clinic?*
3. *What experience do you have of communicating virtually?*
4. *How do you use virtual tools?*
5. *Do you think that there is any advantage / disadvantage to the use of communicating virtually in the care meeting?*
6. *How do you think communication can virtually strengthen your participation and control over your self-care?*

After the Intervention

1. *What experiences do you have after the introduction of virtual meeting in the care meeting?*
2. *Can you describe your positive and negative experiences of using virtual communication in the care meeting?*
3. *Did the technology work in conjunction with virtual care meetings?*
4. *How do you use the virtual contact with diabetes care?*

5. Has virtual communication strengthened your participation and control over your self-care?

6. How do you want to continue with virtual care as a complement to physical meetings?
